# First Estimation of the Spontaneous Mutation Rate in Diatoms

**DOI:** 10.1093/gbe/evz130

**Published:** 2019-06-20

**Authors:** Marc Krasovec, Sophie Sanchez-Brosseau, Gwenael Piganeau

**Affiliations:** 1 Sorbonne Universités, UPMC Univ Paris 06, CNRS, Biologie Intégrative des Organismes Marins (BIOM), Observatoire Océanologique, Banyuls/Mer, France; 2 Department of Plant Sciences, University of Oxford, Oxford, United Kingdom

**Keywords:** spontaneous mutation rate, mutation accumulation, mutation spectrum, *Phaeodactylum tricornutum*, diatoms

## Abstract

Mutations are the origin of genetic diversity, and the mutation rate is a fundamental parameter to understand all aspects of molecular evolution. The combination of mutation–accumulation experiments and high-throughput sequencing enabled the estimation of mutation rates in most model organisms, but several major eukaryotic lineages remain unexplored. Here, we report the first estimation of the spontaneous mutation rate in a model unicellular eukaryote from the Stramenopile kingdom, the diatom *Phaeodactylum tricornutum* (strain RCC2967). We sequenced 36 mutation accumulation lines for an average of 181 generations per line and identified 156 de novo mutations. The base substitution mutation rate per site per generation is *μ*_bs_ = 4.77 × 10^−10^ and the insertion–deletion mutation rate is *μ*_id_ = 1.58 × 10^−11^. The mutation rate varies as a function of the nucleotide context and is biased toward an excess of mutations from GC to AT, consistent with previous observations in other species. Interestingly, the mutation rates between the genomes of organelles and the nucleus differ, with a significantly higher mutation rate in the mitochondria. This confirms previous claims based on indirect estimations of the mutation rate in mitochondria of photosynthetic eukaryotes that acquired their plastid through a secondary endosymbiosis. This novel estimate enables us to infer the effective population size of *P. tricornutum* to be *N*_e_∼8.72 × 10^6^.

## Introduction

**Fig. 1.**
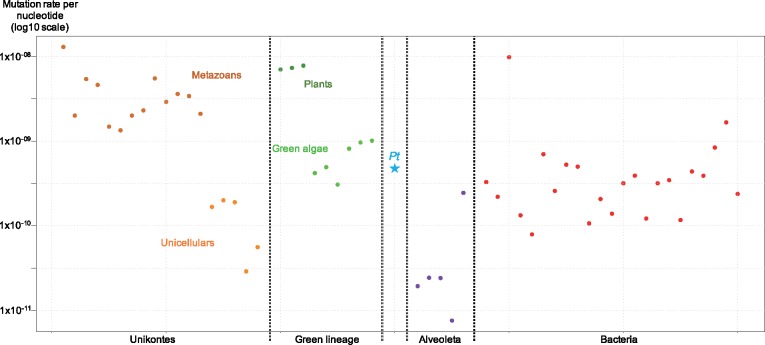
—Spontaneous mutation rates from mutation accumulation and pedigree studies, left to right: *Homo sapiens* (Besenbacher et al. 2016); *Clupea harengus* (Feng et al. 2017); *Mus musculus* (Uchimura et al. 2015); *Ficedula albicollis* (Smeds et al. 2016); *Caenorhabditis elegans, Caenorhabditis briggsae* (Denver et al. 2012); *Pristionchus pacificus* (Weller et al. 2014); *Daphnia pulex* (Flynn et al. 2017); *Drosophila melanogaster* (Schrider et al. 2013); *Heliconius melpomene* (Keightley et al. 2015); *Bombus terrestri, Apis mellifera* (Liu et al. 2017); *Chironomus riparius* (Oppold and Pfenninger 2017); *Saccharomyces cerevisiae* (Zhu et al. 2014); *Schizosaccharomyces pombe* (Farlow et al. 2015); *Dictyostelium discoideum* (Saxer et al. 2012); *Sphaeroforma arctica* (Long et al. 2018); *Arabidopsis thaliana* (Ossowski et al. 2010); *Silene latifolia* (Krasovec et al. 2018); *Prunus persica* (Xie et al. 2016); *Ostreococcus tauri, Ostreococcus mediterraneus, Bathycoccus prasinos, Micromonas pusilla* (Krasovec et al. 2017); *Chlamydomonas reinhardtii* (Ness et al. 2015); *Picochlorum costavermella* (Krasovec et al. 2018); *Pt*: *Phaeodactylum tricornutum*; *Paramecium tetraurelia* (Sung et al. 2012); *Paramecium biaurelia* (Long et al. 2018); *Paramecium sexaurelia* (Long et al. 2018); *Tetrahymena thermophila* (Long et al. 2016); *Plasmodium falciparum* (Hamilton et al. 2017); *Bacillus subtilis* (Sung et al. 2015); *Escherichia coli* (Lee et al. 2012); *Mesoplasma florum* (Sung et al. 2012); *Burkholderia cenocepacia* (Dillon et al. 2015); *Pseudomonas aeruginosa* (Dettman et al. 2016); *Salmonella typhimurium* (Lind and Andersson 2008); *Mycobacterium tuberculosis* (Ford et al. 2011); *Mycobacterium smegmatis* (Kucukyildirim et al. 2016); *Deinococcus radiodurans* (Long et al. 2015); *Vibrio cholerae, Vibrio fischeri* (Dillon et al. 2017); *Ruegeria pomeroyi* (Sun et al. 2017); *Arthrobacter* sp., *Flavobacterium* sp., *Janthinobacterium lividum*, *Micrococcus* sp., *Caulobacter crescentus*, *Rhodobacter sphaeroides*, *Staphylococcus aureus*, *Kineococcus radiotolerans*, *Colwellia psychrerythraea*, *Lactococcus lactis*, *Gemmata obscuriglobus* (Long et al. 2018).

The direct estimation of the spontaneous mutation rate (μ) is one of the most exciting possibilities in evolutionary biology since the development of high-throughput “evolve and resequence” experiments (Katju and Bergthorsson 2019). The spontaneous mutation rate determines the frequency of de novo mutations introduced into a population, allowing adaptation by selection and the renewal of standing genetic variation. Therefore, estimation of μ in a large number of species is necessary to understand the origin of its variation at different scales. Our current knowledge points to a high variation in the mutation rate between species (Lynch et al. 2016; Katju and Bergthorsson 2019). The drift barrier hypothesis is the most accepted explanation of this variation (Sung et al. 2012; Lynch et al. 2016). Under this hypothesis, the mutation load due to deleterious mutations (Charlesworth and Charlesworth 1998) leads to the selection of the lowest possible mutation rate, and species with large *N*_e_ are thus expected to have a lower mutation rate than species with low *N*_e_. Another parameter affecting the mutation rate is the GC nucleotide content. A bias from GC to AT nucleotide mutations has been reported across most branches of the tree of life (Hershberg and Petrov 2010; Ossowski et al. 2010; Denver et al. 2012; Schrider et al. 2013; Krasovec et al. 2017), but the strength of this bias is not equal between species. The difference between the current and the expected GC content reflecting the observed mutation bias between GC and AT nucleotides may explain some of the variation in the mutation rate observed between species (Krasovec et al. 2017) and within a genome (Kiktev et al. 2018). Despite significant advances in understanding the origin of variations in mutation rates, our knowledge is limited to a few biological models. Within eukaryotes, mutation rate estimates are almost exclusively available for organisms in two kingdoms (fig. 1), the Archaeplastida (plants and green algae) and the Unikont (including fungi and metazoans). Outside these two kingdoms, eukaryotic mutation rates are available in Alveolata: in three species from *Paramecium* (*Parameciumtetraurelia*, *Parameciumsexaurelia*, *Parameciumbiaurelia*; Long et al. 2018), in *Tetrahymena thermophila* (Long et al. 2016) and in *Plasmodium falciparum* (Hamilton et al. 2017). In *Paramecium*, the mutation rate is extremely low; one to two orders of magnitude lower than in other unicellular species (fig. 1).

To broaden our knowledge on mutation rate over a wider phylogenetic range, we performed a mutation accumulation (MA) experiment with the model diatom *Phaeodactylum tricornutum* (strain RCC2967 from the Roscoff Culture Collection; Vaulot et al. 2004), belonging to the Stramenopile (or Heterokonta) eukaryotic kingdom. Estimates of the mutation rate of ecological key species such as phytoplankton are scarce and are only available for coastal green algae species (Krasovec et al. 2017, 2018), notably in the model species *Ostreococcus tauri*. Diatoms (Bacillariophyta) are one of the most successful phytoplankton group with a worldwide distribution both in freshwater and marine ecosystems (de Vargas et al. 2015) and comprise about 200,000 species (Mann and Droop 1996). At a global scale, diatoms produce one-fifth of the primary production on Earth and play a fundamental role in carbon and silica bio-geochemical cycles (Falkowski et al. 1998; Kemp et al. 2006; Armbrust 2009; Bowler et al. 2010; Tréguer and De La Rocha 2013) through the long-term carbon sequestration in the sea floor and the production of frustules, silica structures forming the external cell wall of diatoms. After cell death, the frustules sink into the water column and contribute significantly to the formation of siliceous sediments. *Phaeodactylum tricornutum*, and particularly the strain RCC2967 (also known as CCAP1055), originally isolated in 1956 in the North Atlantic Ocean (U.K. coast, Blackpool), is one of the few well studied diatoms species. For decades, it has been used as a model species for studying the evolution of diatoms (Bowler et al. 2008), physiology, and diverse metabolic pathways (Allen et al. 2008; Kroth et al. 2008). Furthermore, several genetic tools have been developed for this species (Zhang and Hu 2014), including CRISPR/Cas9 (Nymark et al. 2016; Serif et al. 2018), and several biotechnological applications are underway (Molina Grima et al. 2003). Its complete genome is diploid with a 27.45 Mb haploid genome size, divided into 33 chromosomes with an average GC content of 49% (Bowler et al. 2008). Estimating the spontaneous mutation rate of a species belonging to the Stramenopile kingdom is essential to expand knowledge about the mutation rate of eukaryotes and brings important insights into phytoplankton diversity and evolution.

## Materials and Methods

### MA Experiment

We performed a MA experiment in liquid medium using the method described by (Krasovec et al. 2016). Briefly, one single cell from the strain RCC2967 (monoclonal culture obtained in 2004 from a harvested strain in 1956; Martino et al. 2007) from the Roscoff Culture Collection (Vaulot et al. 2004) was selected from ten cells sampled from the culture. The ten cells were obtained by pipetting the corresponding volume estimated from the cell concentration by flow cytometry. Resampling six cells out of ten into six wells to choose one well greatly decreases the probability of sampling zero cells (Krasovec et al. 2016). This sampling procedure was repeated to inoculate one single cell for each MA line from this *T*_0_ culture in 24-wells plate in L1 liquid medium with a life cycle of 8 h light–16 h dark at 20°C. Thirty-six MA lines were kept during 98–196 days corresponding to 154–210 generations. Cell concentrations were estimated by flow cytometry at each bottleneck time (every 14 days) to estimate the population size *N*_t_ and to isolate one single cell for reinoculation by dilution. Single cell reinoculation reduces effective population size and thus selection on deleterious spontaneous mutations. *N*_t_ was used to estimate the effective population size (*N*_e_) of MA lines as the harmonic mean of cell number, and to estimate the number of generations (*d*) from the following equation:

(1) $$d=e^{\left[\ln(\frac{N_{t}}{N_{0}})/t\right]}$$

with *t *=* *14 days, *N*_0_=1 cell and *N*_t_ the cell concentration at each bottleneck time.

### Mutation Calling and Spectrum

The genomes of the 36 MA lines and the ancestral *T*_0_ genome were sequenced by Illumina MiSeq technology (125 bp paired-end reads). We checked the raw reads quality with *Trimmomatic* v0.36 (Bolger et al. 2014) and then aligned them to the reference genome (NCBI reference: GCA_000150955.2) using *BWA mem* v0.7.15 with standard parameters (Li and Durbin 2010). Resulting BAM files were treated with *SAMtools* v0.1.19 (Li et al. 2009) and mutation calling was done with and *HaplotypeCaller* from GATK v3.5 following the best practice recommendations (*BaseRecalibrator*, *RealignerTargetCreator*, *IndelRealigner*) (McKenna et al. 2010) and a homemade C code to check the called mutations in the mpileup files. We used similar criteria to identify true de novo mutations as used in previous MA studies in diploid species (Keightley et al. 2015; Krasovec et al. 2018). Several criteria enable to discriminate de novo mutations from heterozygotic SNPs: 1) Callable sites were considered above a threshold of 40 mapping quality (MQ value provided by the vcf GATK file) and 2) a minimum read coverage of 30 and 3) a maximum coverage 2.5 times of the average coverage (to exclude false positives due to repetitive sequences) both in the MA lines and the initial *T*_0_ genome; 4) A de novo mutation was called only if the polymorphism appeared in a unique MA line, was covered by a minimum of ten reads or 30% of the total coverage; 5) each mutation candidate was inspected visually in the mpileup files and in the bam file with IGV (Robinson et al. 2011) both in the MA lines and the *T*_0_ genome; Last, 6) we randomly selected 22 of the final de novo mutation candidates for independent Sanger sequencing in MA lines and in the initial line to validate the candidates. All of the 22 mutations were validated by PCR. Mutation effects (synonymous, nonsynonymous, intergenic) were predicted with *snpEff* v3.6 (Cingolani et al. 2012).

The distribution of the mutations along the genome was compared with the random and independent distributions of mutations with a χ^2^ test. First, we tested the distribution between protein coding, nonprotein coding, intergenic, intronic, exonic, and UTR regions and between chromosomes. Second, we investigated the nucleotide context effect to detect the impact of the previous (5 prime) nucleotide on the probability of mutation (NX). Last, we aligned RNA sequence data from Levering et al. (2017) in standard condition (SRA numbers SRS3629289, SRS3629290, and SRS3629291) to test the correlation between transcription and mutation rates. RNA data were aligned against the reference using STAR with standard parameters (Dobin et al. 2013) and expression level was analyzed with HTSeq (Anders et al. 2015). Statistical analyses were performed with R v3.1.1. The mutation spectrum was obtained using the equations below (Sueoka 1962):

(2) $$R_{1}\text{=}\frac{\left(\text{GC→AT}\right)}{{\mathrm{GC}}_{n}},\;\;R_{2}\text{=}\frac{\left(\text{AT→GC}\right)}{{\mathrm{AT}}_{n}},\;\;R_{3}\text{=}\frac{\left(\text{AT→AT}\right)}{{\mathrm{AT}}_{n}},\;\;R_{4}\text{=}\frac{\left(\text{GC→GC}\right)}{{\mathrm{GC}}_{n}}$$

(3) $${\mathrm{GC}}_{\mathrm{eq}}=\;\frac{R_{2}}{R_{1}+R_{2}}$$

with *GC*_eq_ the GC content of the genome under mutation processes alone. The level of polymorphism of *P. tricornutum* was estimated from the level of heterozygosity of the *T*_0_ genome using the following SNP calling thresholds; a minimum coverage of 20, a minimum mapping quality of 40, and the position defined as diploid (0/1) in the GATK vcf file.

### Mutation Rate Estimation

The mutation rate was calculated for the whole experiment. First, the total number of callable sites was calculated for each MA line using the callable sites quality threshold (see above). The callable site represented ∼98.5% of the genome on average and the average number of generations per line is ∼181, summing up to ∼6,500. Then, the mutation rate was calculated by dividing the total number of mutations by twice the total number of callable sites, as *P. tricornutum* is diploid. The effective population size of the strain was estimated from the equation *π*_s_=4.*N*_e_.μ (Nei and Tajima 1981) with *π*_s_ estimated from the *T*_0_ genome. The *π*_n_/*π*_s_ ratio was estimated from the *T*_0_ genome considering the number of synonymous and nonsynonymous SNPs relative to the synonymous and nonsynonymous callable sites. The number of mitochondria and chloroplast genomes was estimated by dividing the average coverage of each organelle by half the average coverage of the nuclear genome. This calculation inferred ∼11 mitochondria and ∼26 chloroplasts per cell. To deal with heteroplasmy, de novo mutations in organelles were considered true if supported by at least the coverage corresponding to one copy, for example, 1/11 and 1/26 of the total coverage of the mitochondria and the chloroplast, respectively. 

## Results

### Mutation Rates

We applied a previously described MA experiment protocol in liquid medium (Krasovec et al. 2016) to 36 MA lines derived from one ancestral cell. MA lines were maintained over ∼181 generations on average (supplementary table S1, Supplementary Material online) at a low effective population size (*N*_e_∼7) through single cell bottlenecks to ensure minimal selection against deleterious mutations. After sequencing the ancestral and the MA lines, about ∼98.5% of the genome could be screened for mutations. We identified 156 de novo mutations (all leading to heterozygosity, supplementary tables S1 and S2, Supplementary Material online): 151 in the nuclear and 5 in the mitochondrial genome. Within the 86 mutations occurring in protein coding sequences from the nuclear genome, 26 were synonymous, 57 nonsynonymous, and 3 were indels (1 frameshift, 2 codons insertions). The 77 kb mitochondrial genome (35% GC content) contained three mutations (intergenic region) and two indels (codon deletions). No mutations were detected on the 117 kb chloroplast genome (33% GC content). The 22 mutations randomly selected for independent confirmation by Sanger sequencing have all been validated.

The per nucleotide mutation rates (“bs” for base substitutions, “id” for insertion–deletions) are μ_*bs*_=4.77 × 10^−10^ (Poisson CI 95%: 4.04 × 10^−10–^5.61 × 10^−10^) and μ_*id*_=1.58 × 10^−11^ (Poisson CI 95%: 5.13 × 10^−12^–3.69 × 10^−11^) corresponding to a genome wide mutation rate of *U*_*bs*_=0.0125 and *U*_*id*_=0.00041 mutations per haploid genome. The mutation rate in the mitochondria equals μ_*mt_bs*_=1.1 × 10^−9^, μ_*mt_id*_=7.3 × 10^−10^ and is significantly higher than the nuclear mutation rate (χ^2^ test, *P* value < 0.001). The upper limit of the mutation rate of the chloroplast can be estimated to be below 1.0 × 10^−10^ for both μ_*chl_bs*_ and μ_*chl_bs*_. Because mutations are rare events, the mutation distribution between lines is expected to follow a Poisson distribution (Cutler 2000). Otherwise the distribution is considered as overdispersed, which means that the mutation rates between the lines differ significantly. In our data set, the number of mutations per line ranged between 1 and 11 with an average of 4.3 (supplementary fig. S1, Supplementary Material online). This is consistent with a Poisson distribution (χ^2^ test, X-squared = 59.53, df = 63, *P* value = 0.60) with a distribution index of 0.62. Figure 1 shows the nuclear mutation rate of *P. tricornutum* in relation to the genome wide mutation rates available for other species. The data are largely biased toward the two Eukaryote kingdoms, which include the traditional animal and plant model species, highlighting the lack of mutation rate estimates in many lineages.

As *P. tricornutum* is diploid, the rate of heterozygosity along its genome provides an estimation of the level of polymorphism in the population. Single-nucleotide polymorphism (SNP) analysis revealed 239,478 SNPs for 25,613,219 positions, which corresponds to a level of heterozygosity of 0.9%. In protein coding sequences, there are 57,890 synonymous polymorphisms out of 3,479,077 synonymous sites, and 50,925 nonsynonymous polymorphisms out of 11,024,020 nonsynonymous sites, corresponding to *π*_s_=0.0166 *and π*_n_/*π*_s_∼0.278.

### Mutation Spectrum

**Fig. 2.**
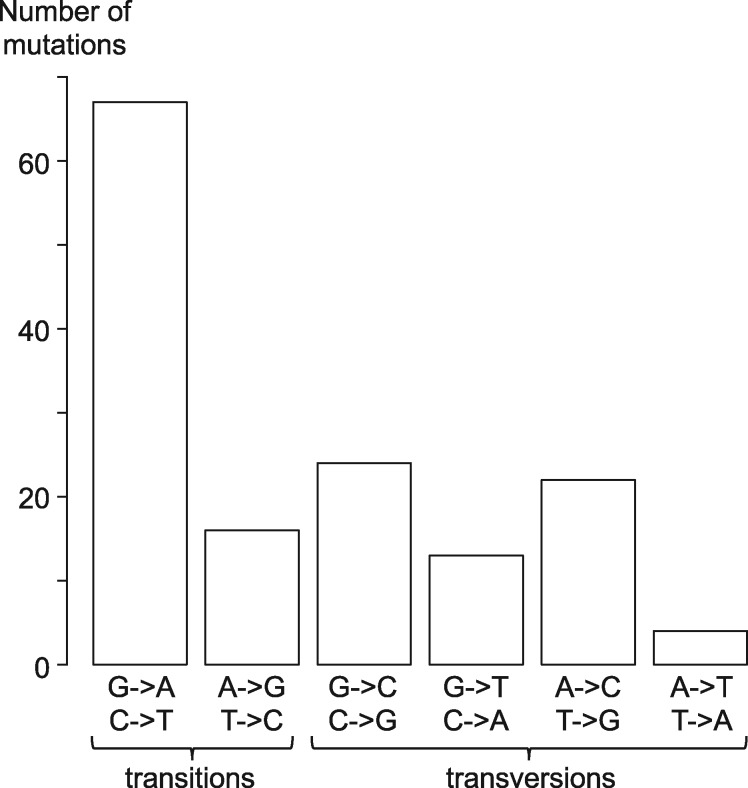
—Distribution of the 146 de novo base substitution mutations in the nuclear genome across the 36 MA lines.

The synonymous and nonsynonymous mutation rates (table 1) did not deviate from the neutral expectation (χ^2^ test, NS), supporting a low selection against nonsynonymous deleterious mutations during the MA experiment. One multiple nucleotide mutation event was detected on chromosome one (three mutations within four nucleotides), which corresponds to ∼0.6% of total mutation events. No bias in the distribution of mutations was observed between protein coding and intergenic sequences (χ^2^ test, NS, table 1), nor between chromosomes (χ^2^ test, NS). Mutated sites had similar RNAseq coverage to constant sites, suggesting that the transcription rate has no effect on the mutation rate in this species (transcription data from Levering et al. 2017). However, there were two biases in the distribution of mutations. First, the dinucleotide context had a significant effect on mutation rates (χ^2^ test, *P* value = 0.0002); CG/CC/TG dinucleotides were mutagenic, while AT/CA/GA/AA displayed a lower mutation rate on the nucleotide at the second position (supplementary fig. S2, Supplementary Material online). Second, there was a mutation bias between G or C and A or T nucleotides (fig. 2): the mutation rate from G or C to A or T was 2.21 times that from A or T to G or C (table 1). Consequently, the expected equilibrium GC content, the GC content resulting from the mutational process alone, equals 31.2%, while the observed GC content is 48.8%. Using a previously described calculation of the expected mutation rate at the equilibrium GC content (Krasovec et al. 2017), the expected mutation rate equals 4.13 × 10^−10^ mutations per site. The difference between the observed GC content and the equilibrium GC content therefore leads to a 15.4% increase in the mutation rate in this species.

**Table 1:** Nuclear Mutation Rates

Substitution	*N*	μ
GC -> AT	80	4.82E-10
AT -> GC	38	2.18E-10
GC -> CG	24	1.44E-10
AT -> TA	4	2.30E-11
Intergenic	57	3.88E-10
Genic		5.00E-10
Intron	5	
Nonsyn	57	
Syn	26	
UTR	3	
Frame shift	1	
Codon insertion	2	

*N*, number of de novo mutations; μ, mutation rate per site per generation.

## Discussion

This first estimation of a spontaneous mutation rate in a species belonging to the Stramenopile kingdom is consistent with previous estimates in four phytoplanktonic species from the Mamiellophyceae class (green algae). It is similar to the spontaneous mutation rates of 4.79 × 10^−10^ mutations per site per generation reported in the model species *O.tauri* (Krasovec et al. 2017). This novel estimation places the average mutation rate of unicellular eukaryotes in the range of 10^−10^ mutations per site per generation, with the notable exception of ciliates (fig. 1).

*Phaeodactylum* *tricornutum*, as well as *O. tauri*, belong to phytoplankton communities isolated in coastal areas in the North Atlantic Ocean and the North West Mediterranean Sea, respectively. Coastal phytoplankton species contribute to primary production supporting the base of the food web in ocean ecosystems. For decades, estuarine and coastal ecosystems have been subjected to high anthropogenic pressures (Jackson et al. 2001), reducing their ecological services like carbon sequestration, raw material source, food, or water purification (Barbier et al. 2011). Knowledge about the mutation rate and the effective population sizes of phytoplankton species is crucial to evaluate and understand their adaptive potential to any selection pressure or environmental change. From the equation *π*_s_=4.*N*_e_.μ and the estimated *π*_s_ value *π*_s_=0.0166, we can deduce the effective population size of *P. tricornutum* RCC2967 is ∼8.7 × 10^6^ (Poisson CI 95%: 7.4 × 10^6^–1.0 × 10^7^). This is similar to the effective population size in *O. tauri N*_e_∼1.2 × 10^7^ (Blanc-Mathieu et al. 2017) one of the rare available estimates in phytoplankton. In diatoms, a second estimate *of N*_e_∼16.5 × 10^7^ has been inferred for the Southern Ocean species *Fragilariopsis cylindrus*, using a mutation rate value of μ=1 × 10^−10^ as a proxy (Mock et al. 2017). Applying the *P. tricornutum* mutation rate, the *F. cylindrus N*_e_ estimation decreases to ∼3.46 × 10^7^. This suggests that the effective population size of *F. cylindrus* is about four times larger than that of *P. tricornutum*. If the effective population size is correlated to the census population in a similar way in these two species, this is consistent with a previous report based on environmental sequencing. Indeed, *Fragilariopsis* has been estimated to be the second most abundant diatom genus after *Chaetoceros* (Malviya et al. 2016) from 46 sampling sites of the TARA Ocean expedition, while *Phaedactylum* could not be detected in this data set. On the contrary, *P. tricornutum* is a littoral species and may thus be expected to have a smaller habitat range. However, the application of macroecological concepts like species ranges on microorganisms, which can reach concentrations of 10^6^ individuals per ml of seawater, has to be interpreted with caution. The effective population size can vary significantly depending on the life cycle and the frequency of sexual reproduction. This has been demonstrated in wild yeast populations (Tsai et al. 2008), where a population with lower sexual reproduction frequency had a smaller effective population size. In diatoms, the primary mode of reproduction is asexual, so that the frequency of the sexual reproduction may also induce a variation in the effective population size, which corresponds to the number of clones in the population. The effective population size is ideally calculated from population genomics data, but such studies are scarce in phytoplankton species. Here, we have estimated the effective population size of *P. tricornutum* from the level of heterozygosity of the strain RCC2967. This strain was isolated in 1956, and a monoclonal culture with fusiform morphotype was generated in 2004 (Martino et al. 2007), and maintained as culture CCAP1055/1 (=RCC2967 or CCMP2561) for 14 years, from which the *T*_0_ line of our MA experiment was derived. This history could have affected the selection of the initial strain and the level of heterozygosity estimated here may not be an accurate estimate of the heterozygosity within the natural population. Lab conditions have their own sources of stress, which may influence the mutation rate. Consistent with this, the microsatellite mutation rate was reported to be higher in recently established lines of cultures of the diatoms species *Pseudo-nitzschia multistriata* (Tesson et al. 2013). Last but not least, the mutation rate can be affected by the environment (Baer 2008) and stress (Jiang et al. 2014). As a consequence, any estimation of experimental mutation rates may differ from the long-term average mutation rate in the natural environment.

### Mutation Rates in Primary and Secondary Organelles

The mitochondrial and chloroplast genomes display a ∼10-fold variation in their mutation rates in *P. tricornutum*. Large variations in organelle mutation rates have been observed in eukaryotes and have been linked to their evolutionary history, in particular in relation to the acquisition of plastids following a primary or secondary endosymbiosis (Smith 2015; Smith and Keeling 2015), phylogenetic constraint, genome structure, and nonadaptive processes (Lynch et al. 2006). Most current data on organellar mutation rates are derived from synonymous substitutions rates (Wolfe et al. 1987; Smith and Keeling 2015). Available evidence from photosynthetic species support that species which evolved after a secondary endosymbiosis event have a higher mitochondrion substitution rate compared with the chloroplast and nuclear mutation rates (Smith and Keeling 2012). Our data on *P. tricornutum* provides direct evidence for a higher mitochondrial mutation rate compared with the chloroplast and nucleus in a microalga evolved from a secondary endosymbiosis event. In addition to this study, direct estimates from MA experiment are available in the green algae *Chlamydomonas reinhardtii* (Ness et al. 2016) and in a few metazoans such as *Caenorhabditis elegans* and *Daphnia pulex* (Denver et al. 2000; Xu et al. 2012; Konrad et al. 2017). Mitochondrial mutation-rate estimates in metazoans are 100 times higher than those for the nuclear genomes in the same lineages (Denver et al. 2000; Xu et al. 2012; Konrad et al. 2017). Conversely, in land plants, the mitochondrial mutation rate is generally lower than the nuclear mutation rate (Drouin et al. 2008). This might be the result of greater accuracy of a specific DNA polymerase involved in organelle replication and repair (Parent et al. 2011; Gualberto et al. 2014). However, this DNA polymerase family is not specific to land plants and the genome of *P. tricornutum* encodes a gene belonging to the same orthologous gene family (DNA polymerase A domain, ORTHO000433 orthologous gene family indexed in the picoPLAZA database; Vandepoele et al. 2013). The three genome compartments seem to have similar mutation rates in green algae, as observed in *Chlamydomonas reinhardtii* (Hua et al. 2012; Ness et al. 2016) and *Volvox carteri* (Smith and Lee 2010). Previous MA experiments on five species of marine green algae did not report any de novo organellar mutations (Krasovec et al. 2017, 2018). Given the mitochondrial and chloroplast genome sizes in *O. tauri* (Mamiellophyceae), it follows μ are <8.28 × 10^−10^ and 1.45 × 10^−9^ in the chloroplast and the mitochondria, respectively. The application of the same reasoning to *Picochlorum costavermella* (Trebouxiophyceae) leads to the conclusion that the mutation rates are <8.43 × 10^−9^ and 1.8 × 10^−8^ in the chloroplast and the mitochondria for a nuclear mutation rate of 1.01 × 10^−9^ mutations per site. These maximum organellar mutation rate estimates would be consistent with a higher mutation rates in organelles than in the nuclear genomes in these lineages. Precise estimations, implying MA experiments over a higher number of generations are obviously needed to test this hypothesis.

### Mutation Rate Variation

The difference between the observed GC and the equilibrium GC content has a moderate effect on the mutation rate (+15.4%) in *P. triconutum*. This effect is variable between species; in *Arabidopsisthaliana* and *Chlamydomonasreinhardtii*, it leads to a mutation rate increase of 64% and 42%, respectively (see table S12 of Krasovec et al. 2017). In the bacteria *Mesoplasma florum*, which appears to have the highest mutation rate measured in bacteria (fig. 1), the GC bias effect is very high with an observed μ ∼263% its expected value at equilibrium GC content. Within the Mamiellophyceae, the mutation rate is increased by ∼2 to ∼12% depending on the species. The difference between the observed and equilibrium GC content may be explained by three processes, either a recent change in the mutation bias between AT and GC nucleotides, or GC biased gene conversion (Duret and Galtier 2009), or selection on GC (Eyre-Walker 1999; Hildebrand et al. 2010). A change in the mutation bias may be due to a change in the rate of oxidation of the G nucleotide to 8-oxoguanine (Cooke et al. 2003) or a change in the proportion of C nucleotide methylation, which has an increased probability of mutation toward T (Holliday and Grigg 1993). Cytosine methylation has been documented in *P. tricornutum*, where ∼6% of the genome is methylated (Huff and Zilberman 2014; Veluchamy et al. 2014). This may explain some of the GC bias, even moderate, observed in this species.

The mutation rate of *P. tricornutum* is not homogeneous within the genome. The effect of the nucleotide context has already been observed in others species such as the green algae *Chlamydomonas reinhardtii* (Ness et al. 2015) and the bacteria *Bacillus subtilis* (Sung et al. 2015). The link between mutation probability and nucleotide context is of primary importance for molecular evolution on synonymous versus nonsynonymous sites. In the bacteria *Bacillus subtilis* (Sung et al. 2015), the differences in the probability of mutation between sites may induce a difference in the rate of evolution between codons. In *P. tricornutum*, the most mutable dinucleotide is CG->CN. A codon starting with CG codes for the amino acid arginine: this amino acid therefore has a higher probability of mutating into another amino acid. Since G undergoes more frequent mutations than A, it is expected that arginine codons will be more frequently replaced by histidine or glutamine. Similarly, codons ending in CG (serine, proline, alanine, and threonine) will evolve faster than synonymous codons ending with GA. In conclusion, knowledge of the mutation spectrum is not only essential to predict the rate of adaptation to environmental change but also to infer the evolutionary history through accurate calibration of the molecular clock.

## Supplementary Material

Supplementary data are available at *Genome Biology and Evolution* online.

## Supplementary Material

Supplementary_Material_evz130

## Notes

**Data deposition:** This project has been deposited at NCBI under the accession PRJNA478011.
