# Supplementary figures and images for: First Estimation of the Spontaneous Mutation Rate in Diatoms
Source: Genome Biol Evol. 2019 Jun 20;11(7):1829–37. doi: 10.1093/gbe/evz130 (PMC6604790; doi:10.1093/gbe/evz130)

Mutation rate per  
nucleotide

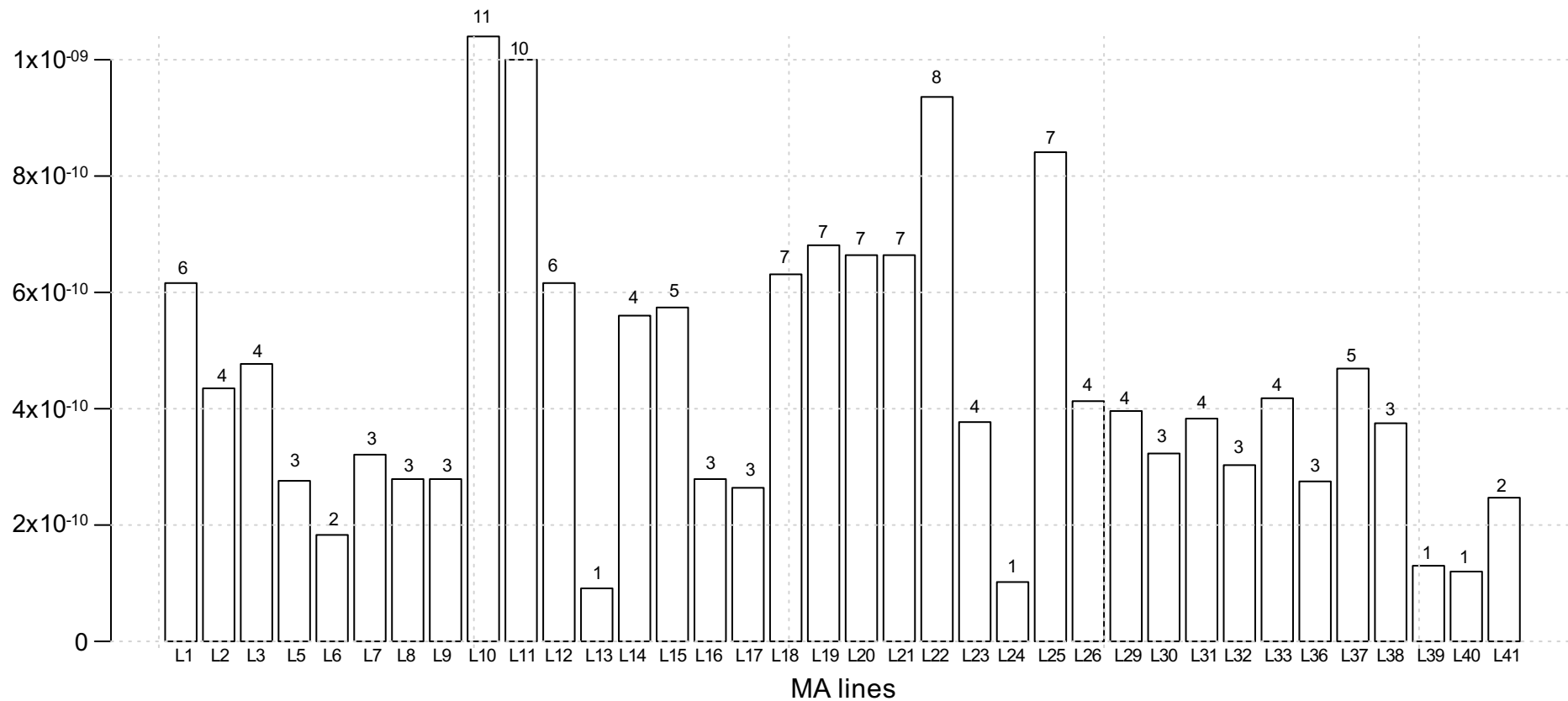

Supplement: Supplementary_Material_evz130 [file supplementary_material_evz130.zip › Figure_S1.pdf]

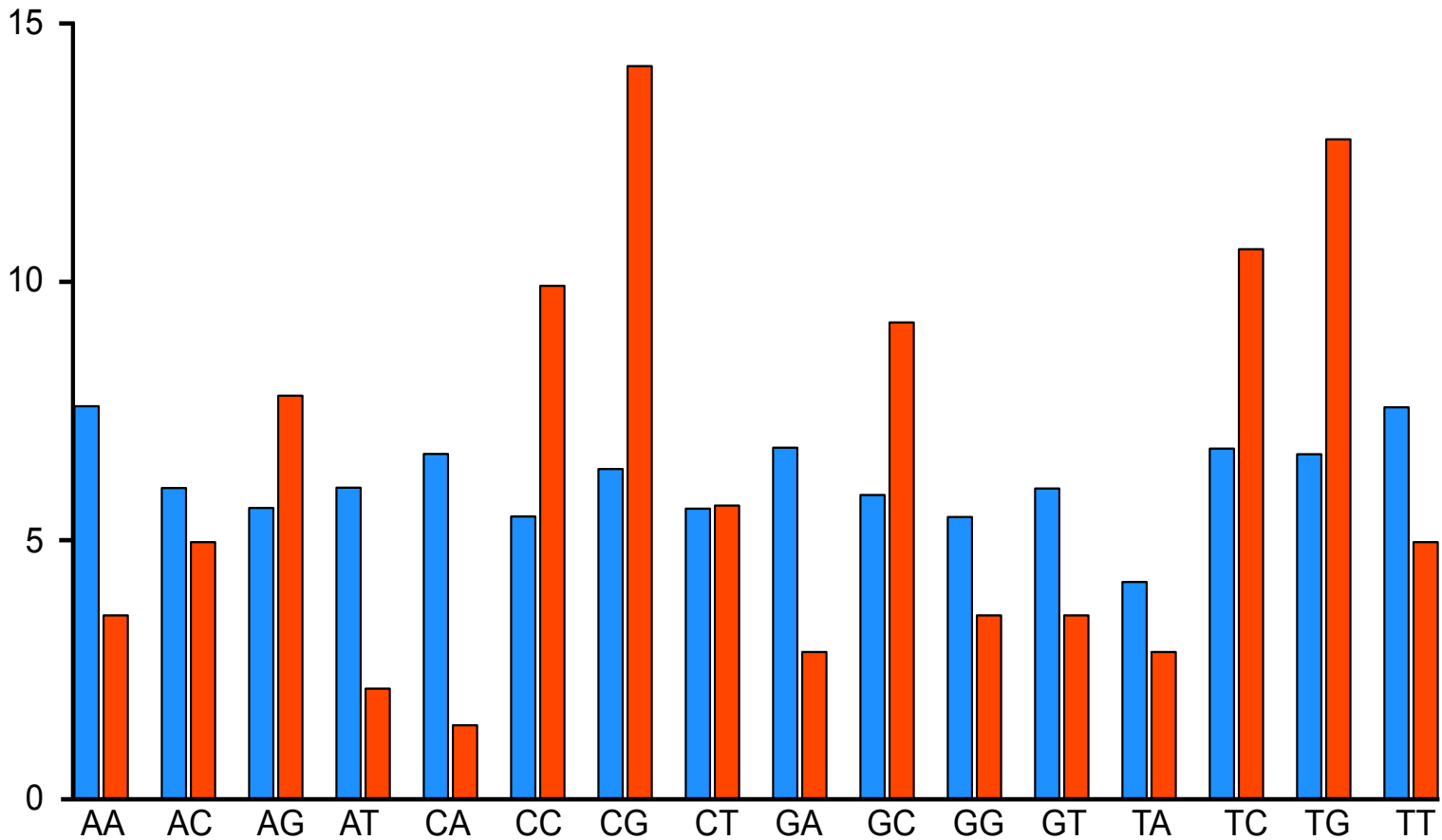

Supplement: Supplementary_Material_evz130 [file supplementary_material_evz130.zip › Figure_S2.pdf]
