# Supplementary material for: First Estimation of the Spontaneous Mutation Rate in Diatoms
Source: Genome Biol Evol. 2019 Jun 20;11(7):1829–37. doi: 10.1093/gbe/evz130 (PMC6604790; doi:10.1093/gbe/evz130)
Supplement: Supplementary_Material_evz130 [file supplementary_material_evz130.zip › SUPPLEMENTARY_FILE.doc]

**SUPPLEMENTARY DATA**

**Table S1.** Data summary per line.

**Table S2.** The 156 mutations identified in the whole MA experiment.

**Figure S1.** Mutation rate of each MA line.

**Figure S2.** Proportion of dinucleotides.

**Table S1.** Data summary per line. *Gb* is the amount of data, *G** is the callable genome with coverage average in brackets, *Gen* is the number of generations of the line, *Sub* and *InDel* are the number of substitutions and insertion-deletions per line.

| **Line** | **Biosample** | **Gb** | **G*** | **Gen** | **Sub** | **InDel** |
| --- | --- | --- | --- | --- | --- | --- |
| Initial_line (T_0_) | SAMN09489425 | 2.48 | 98.5 (95.3) | - | - | - |
| MA_line_L1 | SAMN09489399 | 1.4 | 98.6 (94.1) | 180 | 6 | - |
| MA_line_L10 | SAMN09489389 | 3.11 | 98.5 (207.5) | 195 | 11 | - |
| MA_line_L11 | SAMN09489390 | 1.24 | 98.7 (87.4) | 185 | 10 | - |
| MA_line_L12 | SAMN09489391 | 1.25 | 98.5 (58.6) | 180 | 6 | - |
| MA_line_L13 | SAMN09489392 | 1.83 | 98.5 (71.4) | 203 | 1 | - |
| MA_line_L14 | SAMN09489393 | 1.18 | 98.2 (95.0) | 132 | 4 | - |
| MA_line_L15 | SAMN09489394 | 0.78 | 98.5 (56.7) | 161 | 5 | - |
| MA_line_L16 | SAMN09489395 | 1.62 | 97.1 (87.6) | 199 | 2 | 1 |
| MA_line_L17 | SAMN09489396 | 2 | 98.5 (85.6) | 210 | 2 | 1 |
| MA_line_L18 | SAMN09489397 | 0.98 | 98.5 (57.6) | 205 | 7 | - |
| MA_line_L19 | SAMN09489398 | 2.97 | 96.7 (185.9) | 190 | 7 | - |
| MA_line_L2 | SAMN09489408 | 1.36 | 98.5 (104.3) | 170 | 4 | - |
| MA_line_L20 | SAMN09489400 | 1.3 | 98.5 (110.8) | 195 | 6 | 1 |
| MA_line_L21 | SAMN09489401 | 1.69 | 98.6 (93.5) | 195 | 7 | - |
| MA_line_L22 | SAMN09489402 | 1.45 | 98.5 (107.3) | 158 | 8 | - |
| MA_line_L23 | SAMN09489403 | 2.61 | 98.6 (184.6) | 196 | 2 | 2 |
| MA_line_L24 | SAMN09489404 | 1.7 | 98.7 (112.8) | 182 | 1 | - |
| MA_line_L25 | SAMN09489405 | 1.72 | 86.8 (33.8) | 154 | 7 | - |
| MA_line_L26 | SAMN09489406 | 3.08 | 98.6 (210.7) | 179 | 3 | 1 |
| MA_line_L29 | SAMN09489407 | 1.3 | 98.0 (99.1) | 187 | 4 | - |
| MA_line_L3 | SAMN09489417 | 1.33 | 98.7 (105.6) | 155 | 4 | - |
| MA_line_L30 | SAMN09489409 | 1.37 | 98.5 (108.8) | 172 | 3 | - |
| MA_line_L31 | SAMN09489410 | 2.88 | 98.5 (192.5) | 193 | 4 | - |
| MA_line_L32 | SAMN09489411 | 1.43 | 98.7 (109.0) | 183 | 3 | - |
| MA_line_L33 | SAMN09489412 | 5.01 | 98.6 (350.1) | 177 | 4 | - |
| MA_line_L36 | SAMN09489413 | 1.32 | 98.7 (99.9) | 202 | 3 | - |
| MA_line_L37 | SAMN09489414 | 2.08 | 98.5 (121.1) | 197 | 5 | - |
| MA_line_L38 | SAMN09489415 | 2.58 | 98.6 (157.1) | 148 | 3 | - |
| MA_line_L39 | SAMN09489416 | 3.96 | 98.6 (305.8) | 142 | 1 | - |
| MA_line_L40 | SAMN09489418 | 1.72 | 98.5 (93.9) | 154 | 1 | - |
| MA_line_L41 | SAMN09489419 | 1.54 | 98.6 (124.0) | 150 | 2 | - |
| MA_line_L5 | SAMN09489420 | 1.29 | 98.4 (83.1) | 201 | 3 | - |
| MA_line_L6 | SAMN09489421 | 1.71 | 98.5 (91.0) | 202 | 1 | 1 |
| MA_line_L7 | SAMN09489422 | 1.79 | 98.6 (136.8) | 173 | 3 | - |
| MA_line_L8 | SAMN09489423 | 1.42 | 98.6 (104.1) | 199 | 3 | - |
| MA_line_L9 | SAMN09489424 | 1.2 | 98.5 (98.9) | 199 | 3 | - |

**Table S2. The 156 mutations identified in the whole MA experiment. *Int*: intergenic. *Syn*: synonymous substitution. *Non* *Syn*: non-synonymous substitution.**

| **Chromosome** | **Position** | **Reference** | **Mutation** | **Effect** |
| --- | --- | --- | --- | --- |
| chromosome01 | 45826 | T | C | Int |
| chromosome01 | 134796 | C | T | Syn |
| chromosome01 | 315714 | C | T | Int |
| chromosome01 | 346673 | C | G | Int |
| chromosome01 | 381781 | C | T | Non Syn |
| chromosome01 | 515873 | G | A | Non Syn |
| chromosome01 | 605402 | G | A | Intron |
| chromosome01 | 865078 | A | C | Int |
| chromosome01 | 1260808 | T | G | Int |
| chromosome01 | 1323198 | T | G | Non Syn |
| chromosome01 | 1323199 | A | T | Syn |
| chromosome01 | 1323201 | C | T | Non Syn |
| chromosome01 | 1396958 | G | A | Int |
| chromosome01 | 1461041 | G | C | Non Syn |
| chromosome01 | 1525199 | G | C | Non Syn |
| chromosome01 | 1611691 | C | G | Non Syn |
| chromosome01 | 1662336 | T | C | Non Syn |
| chromosome01 | 1694254 | G | A | Int |
| chromosome01 | 1740545 | A | C | Int |
| chromosome01 | 1742136 | G | A | Syn |
| chromosome01 | 1810472 | C | T | Syn |
| chromosome01 | 1920739 | G | T | Stop Gained |
| chromosome01 | 1972278 | T | G | Non Syn |
| chromosome01 | 2072616 | C | T | 5' UTR |
| chromosome01 | 2092144 | G | A | Int |
| chromosome01 | 2130231 | T | G | Int |
| chromosome01 | 2148411 | C | T | Non Syn |
| chromosome02 | 302290 | G | A | Non Syn |
| chromosome02 | 433451 | G | A | Syn |
| chromosome02 | 724247 | T | C | Int |
| chromosome02 | 770885 | C | A | Syn |
| chromosome02 | 852842 | G | C | Non Syn |
| chromosome02 | 853465 | C | T | Syn |
| chromosome02 | 1060404 | T | C | Syn |
| chromosome02 | 1256129 | G | A | Int |
| chromosome03 | 328 | G | A | Syn |
| chromosome03 | 124005 | G | C | Int |
| chromosome03 | 194753 | G | C | Non Syn |
| chromosome03 | 530402 | G | A | Syn |
| chromosome03 | 672466 | C | T | Non Syn |
| chromosome03 | 871029 | G | A | Syn |
| chromosome03 | 1032632 | G | A | Non Syn |
| chromosome03 | 1139044 | G | T | Intron |
| chromosome03 | 1208246 | C | A | Int |
| chromosome03 | 1279767 | G | C | Int |
| chromosome03 | 1345265 | G | C | Int |
| chromosome03 | 1368439 | A | C | Int |
| chromosome03 | 1447044 | G | A | Int |
| chromosome04 | 224078 | C | G | Non Syn |
| chromosome04 | 293989 | C | T | Int |
| chromosome04 | 346860 | C | A | Int |
| chromosome04 | 376671 | A | G | Int |
| chromosome04 | 489826 | C | T | Syn |
| chromosome04 | 630425 | C | G | Int |
| chromosome04 | 660703 | C | A | Int |
| chromosome04 | 665621 | A | T | Syn |
| chromosome04 | 743586 | C | G | Non Syn |
| chromosome04 | 1232654 | T | G | Int |
| chromosome06 | 34695 | A | T | Non Syn |
| chromosome06 | 56691 | G | T | Int |
| chromosome06 | 139959 | T | G | Int |
| chromosome06 | 179834 | A | G | Non Syn |
| chromosome06 | 632305 | T | G | Non Syn |
| chromosome06 | 779261 | T | G | Int |
| chromosome06 | 1034666 | A | C | Int |
| chromosome07 | 294789 | G | A | Non Syn |
| chromosome07 | 573907 | C | G | Int |
| chromosome07 | 591850 | T | G | Non Syn |
| chromosome07 | 634018 | C | T | Non Syn |
| chromosome07 | 731951 | C | T | Non Syn |
| chromosome07 | 839759 | G | A | Stop Gained |
| chromosome08 | 15060 | G | A | Non Syn |
| chromosome08 | 455503 | T | C | Non Syn |
| chromosome08 | 572183 | G | C | Non Syn |
| chromosome08 | 666492 | A | G | Non Syn |
| chromosome08 | 690710 | C | G | Non Syn |
| chromosome08 | 775293 | G | A | Syn |
| chromosome09 | 316930 | C | G | Int |
| chromosome09 | 349577 | G | C | Non Syn |
| chromosome09 | 560486 | C | T | Non Syn |
| chromosome09 | 776209 | G | A | Int |
| chromosome09 | 940112 | C | G | Int |
| chromosome10 | 46066 | C | T | Non Syn |
| chromosome10 | 241724 | C | T | Int |
| chromosome10 | 380494 | G | A | Int |
| chromosome10 | 489230 | G | T | Int |
| chromosome10 | 492570 | C | T | Intron |
| chromosome10 | 616753 | C | T | Non Syn |
| chromosome10 | 905354 | T | G | Int |
| chromosome11 | 136627 | G | A | Non Syn |
| chromosome11 | 254596 | A | G | Non Syn |
| chromosome11 | 340920 | C | T | Syn |
| chromosome12 | 14955 | A | G | Int |
| chromosome12 | 412665 | C | T | Non Syn |
| chromosome13 | 394168 | T | C | Non Syn |
| chromosome13 | 490755 | T | G | Non Syn |
| chromosome13 | 514026 | C | T | Stop Gained |
| chromosome14 | 8939 | C | T | 3' UTR |
| chromosome14 | 313844 | A | C | Int |
| chromosome14 | 322974 | G | A | Non Syn |
| chromosome14 | 391378 | G | T | Non Syn |
| chromosome14 | 670693 | T | G | Non Syn |
| chromosome14 | 712413 | C | A | Stop Gained |
| chromosome15 | 151222 | A | C | Int |
| chromosome15 | 484832 | C | T | Int |
| chromosome15 | 509530 | A | C | Syn |
| chromosome15 | 604567 | A | G | 5' UTR |
| chromosome16 | 188415 | C | A | Int |
| chromosome16 | 194286 | G | A | Int |
| chromosome16 | 200840 | A | T | Non Syn |
| chromosome16 | 402860 | G | A | Syn |
| chromosome17 | 24742 | C | G | Int |
| chromosome17 | 244015 | G | A | Non Syn |
| chromosome17 | 321558 | T | C | Syn |
| chromosome18 | 281472 | G | A | Int |
| chromosome18 | 341193 | G | A | Syn |
| chromosome18 | 476889 | C | T | Syn |
| chromosome18 | 487193 | C | G | Intron |
| chromosome19 | 47726 | G | A | Non Syn |
| chromosome19 | 60611 | G | C | Non Syn |
| chromosome19 | 212837 | G | A | Int |
| chromosome19 | 251555 | C | T | Int |
| chromosome19 | 251597 | G | A | Int |
| chromosome20 | 273033 | C | T | Int |
| chromosome20 | 352487 | T | C | Non Syn |
| chromosome20 | 370168 | C | T | Non Syn |
| chromosome20 | 665780 | G | A | Int |
| chromosome21 | 100385 | G | C | Non Syn |
| chromosome22 | 15478 | C | T | Non Syn |
| chromosome22 | 85244 | G | A | Intron |
| chromosome22 | 133490 | C | T | Int |
| chromosome22 | 468799 | G | T | Non Syn |
| chromosome23 | 139360 | T | G | Non Syn |
| chromosome23 | 139367 | A | G | Non Syn |
| chromosome23 | 139374 | T | C | Syn |
| chromosome23 | 139379 | G | A | Syn |
| chromosome23 | 139415 | T | G | Syn |
| chromosome23 | 231480 | C | G | Syn |
| chromosome24 | 12069 | G | A | Non Syn |
| chromosome25 | 231314 | C | T | Int |
| chromosome25 | 308188 | C | T | Syn |
| chromosome26 | 207740 | G | A | Syn |
| chromosome28 | 160906 | G | C | Int |
| chromosome28 | 384941 | C | A | Int |
| chromosome29 | 297872 | G | A | Int |
| chromosome33 | 77833 | A | C | Int |
| chromosome01 | 571575 | CACTGTCGTAT | C | Frame Shift |
| chromosome01 | 2420592 | CA | C | Int |
| chromosome01 | 2491725 | T | TGGC | Codon Insertion |
| chromosome06 | 939723 | G | GCCTGCCTA | Int |
| chromosome09 | 844451 | T | TACA | Codon Insertion |
| mitochondrion | 10513 | AT | A | Codon Deletion |
| mitochondrion | 7640 | CTAGCATCTTACATTT | C | Codon Deletion |
| mitochondrion | 53976 | C | T | Int |
| mitochondrion | 54379 | C | T | Int |
| mitochondrion | 65912 | G | A | Int |

**Figure S1.** Mutation rate of each MA line with the number of mutations above each bar.

**Figure S2.** Proportion of dinucleotide in the genome and mutation distribution considering mutation at the second positions. In blue is the frequency in the genome, in red is the mutation distribution.
